# Defining Molecular Sensors to Assess Long-Term Effects of Pesticides on Carcinogenesis

**DOI:** 10.3390/ijms150917148

**Published:** 2014-09-25

**Authors:** Fanny L’Héritier, Maud Marques, Myriam Fauteux, Luc Gaudreau

**Affiliations:** Département de Biologie, Université de Sherbrooke, 2500 boul. de l’Université, Sherbrooke, QC J1K 2R1, Canada; E-Mails: Fanny.Lheritier@USherbrooke.ca (F.L.); Maud.Marques@USherbrooke.ca (M.M.); Myriam.Fauteux@USherbrooke.ca (M.F.)

**Keywords:** aryl hydrocarbon receptor (AhR), ERα, pesticides

## Abstract

The abundance of dioxins and dioxin-like pollutants has massively increased in the environment due to human activity. These chemicals are particularly persistent and accumulate in the food chain, which raises major concerns regarding long-term exposure to human health. Most dioxin-like pollutants activate the aryl hydrocarbon receptor (AhR) transcription factor, which regulates xenobiotic metabolism enzymes that belong to the cytochrome P450 1A family (that includes *CYP1A1* and *CYP1B1*). Importantly, a crosstalk exists between estrogen receptor α (ERα) and AhR. More specifically, ERα represses the expression of the *CYP1A1* gene, which encodes an enzyme that converts 17β-estradiol into 2-hydroxyestradiol. However, (ERα) does not repress the *CYP1B1* gene, which encodes an enzyme that converts 17β-estradiol into 4-hydroxyestradiol, one of the most genotoxic estrogen metabolites. In this review, we discuss how chronic exposure to xenobiotic chemicals, such as pesticides, might affect the expression of genes regulated by the AhR–ERα crosstalk. Here, we focus on recent advances in the understanding of molecular mechanisms that mediate this crosstalk repression, and particularly on how ERα represses the AhR target gene *CYP1A1*, and could subsequently promote breast cancer. Finally, we propose that genes implicated in this crosstalk could constitute important biomarkers to assess long-term effects of pesticides on human health.

## 1. Introduction

The aryl hydrocarbon receptor (AhR) is a ligand-activated transcription factor that belongs to the basic helix–loop–helix (bHLH)/Per–Arnt–Sim (PAS) family [1]. It is maintained inactive in the cytoplasm in a complex with the Hsp90–XAP2–p23 chaperones [2]. Activation of AhR occurs via direct binding of its ligands, where it translocates into the nucleus to form a complex with the bHLH/PAS Arnt protein. This heterodimer complex then binds regulatory consensus regions termed xenobiotic response elements (XREs) located at proximity to its target genes, which include the phase I detoxifying monooxygenases *CYP1A1* and *CYP1B1* [3]. The best-characterized AhR ligands commonly fall into the following classes: halogenated aromatic hydrocarbons (HAHs), such as 2,3,7,8-tetrachlorodibenzo-*p*-dioxin (TCDD), polycyclic aromatic hydrocarbons (PAHs) and polychlorinated biphenyls (PCBs) [4]. All these chemicals are human by-products that emanate from human-based activities. For example, high levels of PAHs are found in barbecue cooked meat, and are becoming a major concern for human health considering their persistence in the environment.

AhR is best known for its role as a mediator of toxicity during environmental pollutant metabolism. However, recent studies show that AhR plays an important role in normal physiology and development as well [5,6,7,8,9]. There has also been various studies demonstrating interactions and crosstalk between AhR and various intracellular signaling pathways, including NF-κB [10,11], Nrf2 [12], Rb/E2F [13], and Sp1 [14], as well as other transcription factors belonging to the nuclear receptor family, such as the androgen receptor [15], and the estrogen receptors (ERα and ERβ ) [15,16,17,18,19]. The crosstalk between the signaling pathways of AhR and ERα receptors will be the main focus of this review, especially in the context of breast cancer development.

AhR is proposed to have a role in breast development *in utero*, during pregnancy and also as previously mentioned, in breast cancer initiation. The mammary gland is composed of different cell types and structures that are subject to change during various and specific life stages, *i.e.*, puberty and pregnancy. Consequently, exposure to AhR agonists could have different outcomes making it challenging to show direct effects of pollutants on breast cancer incidence. Several studies reported higher AhR expression in more malignant breast cancer cell lines as well as a positive correlation with breast tumors aggressiveness [20,21]. However, another study showed that 72.7% of benign mammary tissues have nuclear AhR immunohistochemistry staining [22]. The two main target genes of AhR in breast tissues are *CYP1A1* and *CYP1B1*. CYP1A1 mRNA and protein levels are low in normal and breast tumor tissues. However, higher CYP1B1 mRNA and protein levels are observed in breast tumors as compared to normal tissues.

## 2. Ligands and Agonists of Aryl Hydrocarbon Receptor (AhR)

The best-characterized high-affinity AhR ligands are hydrophobic molecules that bear aromatic carbon rings. However, there is more and more evidence suggesting that there is a growing spectrum of structurally diverse chemicals that are capable of binding and/or activating the AhR signaling pathway. In this section, well will briefly discuss known endogenous and exogenous ligands of AhR, with an emphasis on pesticides.

## 3. Endogenous Ligands

Human exposure to toxic environmental chemicals has greatly changed in the last 200 years, especially in societies struck by the industrial revolution. On the one hand, this suggests that AhR has probably been the target of recent evolutionary pressure in various species. One example recently published by Mark Hahn’s laboratory [23] showed that Atlantic tomcod from the Hudson River exposed to high level of PCB, released by General Electric facilities, exhibited a variant of the *AHR2* gene, which is absent in nearly all tomcod from elsewhere. This variant possesses less affinity for TCDD and others AhR ligands, and consequently has less transcriptional activity and toxicity. On the other hand, it is reasonable to assume that AhR response to these man-made chemicals is a sign of biochemical versatility of the receptor to bind a wide range of molecules (see below). This, in turn, may suggest that AhR could also respond to various endogenous ligands, *i.e.*, produced by the cells themselves. Studies showing that the AhR signaling pathway is active in the absence of exogenous ligands have reinforced this view, and lead scientists to suspect a role for AhR in physiological functions of cells. Consequently, intensification of research to find endogenous AhR ligands has sparked over the last few years. The following chemicals, which are endogenously synthesized by human tissues, are known activators of AhR: indigoids, equilenin, heme metabolites, arachidonic acid metabolites, eicosanoids, and tryptophan derivatives [24,25,26]. l-Kynurenine, a tryptophan catabolite, was shown to activate AhR and to be constitutively produced by tumor cells, and astonishingly promote their survival and escape from the immune system [27]. The later observations provide new evidence that an endogenous ligand of AhR promotes carcinogenesis, thereby conferring oncogenic properties to AhR.

## 4. Exogenous Ligands

### 4.1. “Classical” Synthetic AhR Ligands

As mentioned earlier, AhR is activated by a large group of environmental pollutants composed of HAHs (including TCDD), PAHs (including benzo[α]pyrene), and PCBs [4]. The majority of these chemicals are formed and released as by-products of human activities, mostly emanating from industrial and combustion processes [28].

### 4.2. Natural/Dietary Compounds

One of the most obvious potential sources of naturally occurring AhR ligands probably comes from our diet. The non-toxic indole-3-carbinol (I3C) and its derivatives, including 3,3'-diindolylmethane (DIM), have been gathering great attention lately for their anticancer properties. However, there are other natural compounds that have been reported to activate the AhR signaling pathway, including flavonoids, carotenoids, curcumin, and others, which are reviewed in Nguyen *et al.* and Stejskalova *et al.* [24,26].

### 4.3. Pesticides

In depth analysis of a wide range of pesticides currently used in agriculture revealed that many of them possess a structure very similar to well-known AhR ligands, such as TCDD. Considering that exposure to pesticides may lead to various human diseases, including cancer, the implication of AhR in this process should be carefully assessed. It has been documented for quite some time now that pesticides possess endocrine disrupting properties [29]. Several currently used pesticides have been reported to have estrogenic activity [30,31,32,33,34,35,36]. Endocrine disruption of the estrogen receptor-signaling pathway can be direct (*i.e.*, chemicals bind the estrogen receptor and modulate its activity) or indirect (*i.e.*, chemicals affect another pathway such as the AhR case, which then modulates ERα activity). More recently, Kojima *et al.* used *in vitro* reporter gene assays to screen 200 pesticides for estrogen and androgen activities. They also tested the AhR agonistic activity for these 200 pesticides. Out of those, three herbicides—propanil, linuron and diuron—showed potent AhR agonistic activity and only two—chlorpyrifos and isoxathion—showed both AhR and ERα activities [37,38].

Several studies were able to measure pesticide concentrations in breast cancer biopsies (adipose tissues and tumor sections). As such, Cassidy and collaborators showed that heptachlor epoxide induces nitric oxide production in the breast tissues, which may contribute to tumor initiation by increasing DNA damages in the cells [39]. Another group evaluated breast cancer risk and exposure to environmental estrogens—more specifically, 16 organochlorine pesticides. The authors found that the presence of a higher concentration of the pesticides aldrin and lindane is associated with an increased risk of developing breast cancer, especially for leaner postmenopausal women. [40] All these studies suggest an important role for pesticides and others environmental chemicals in the initiation and development of breast cancer; however, none of the studies thus far elude to mechanisms of action of these chemical, and how they affect the ERα and AhR signaling pathways (or their crosstalk).

**Figure 1:**
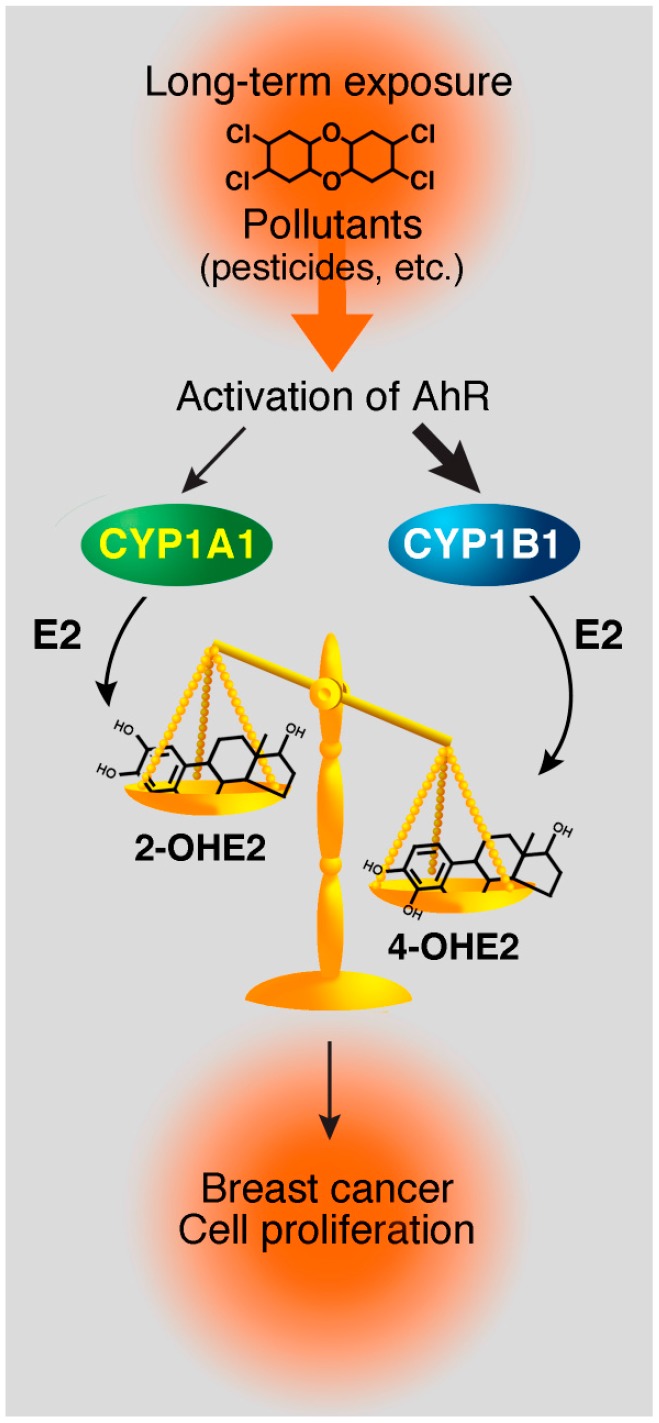
Proposed model for initiation of breast cancer by pollutants and pesticides. Long-term exposure to pollutants and pesticides, which could induce the aryl hydrocarbon receptor (AhR) and the estrogen receptor α (ERα) signaling pathways, will create an imbalance between CYP1A1 and CYP1B1 enzymes. Thus, this will modify the 2-OHE2/4-OHE2 ratio and could contribute to mammary carcinogenesis.

Certain pesticides are capable of activating AhR and consequently inducing the expression of *CYP1A1* and *CYP1B1* genes. The enzymes encoded by these two genes are involved in 17β-estradiol (E2) metabolism: CYP1A1 and CYP1B1 convert E2 into 2‑hydroxyestradiol (2-OHE2) and 4-hydroxyestradiol (4-OHE2), respectively [41,42]. Several studies have compared the tumorigenic potential of E2 and its metabolites, such as 2-OHE2 and 4-OHE2 [43,44,45]. In fact, 2-OHE2 inhibits cellular growth of breast cancer cell lines [46], and induces apoptosis in immortalized mammary cells [47]. In contrast, 4-OHE2 induces kidney tumors in Syrian hamsters [45], and enhances proliferation and mutagenesis by promoting the formation of depurinated adducts on DNA [48]. 4-OHE2 is reported to be one of the most genotoxic estradiol metabolites. It has also been reported that breast cancer cells metabolize more 4-OHE2 than normal cells [49]. Thus, the ratio between 2-OHE2/4-OHE2 metabolites, and consequently, the ratio between CYP1A1/CYP1B1 enzymes, appears to be important in the initiation of carcinogenesis in mammary tissues. In addition, if some pesticides induce more *CYP1B1* expression than *CYP1A1*, this could also lead to imbalances in the ratio between CYP1A1 and CYP1B1 enzymes. Consequently, if we consider this scenario on a long-term exposure scale, more 4-OHE2 metabolites would be formed, hence leading to the accumulation of DNA adducts and potentially initiating cancer development (Figure 1). Estrogen metabolites have already been established as potential biomarkers for susceptibility to breast cancer [50]. Thus, linking pesticide exposure to this imbalanced ratio in favor of the CYP1B1 enzyme would provide direct evidence that pesticides may play a role in the early steps of breast cancer development.

It should also be taken into consideration that some of these pesticides probably contain impurities, and may even be contaminated with dioxins, which could only aggravate their adverse effects. The most notorious example is Agent Orange, an herbicide used during the Vietnam War by the U.S. military as a defoliating product. The now-banned herbicide was a mixture of 2,4-D (2,4-dichlorophenoxyacetic acid) and 2,4,5-T (2,4,5-trichlorophenoxyacetic acid), and it was later shown that 2,4,5-T was contaminated with small amounts of TCDD [51]. In 2004, the latest update of the Veterans and Agent Orange report of the National Academies’ Institute of Medicine (IOM) claimed there was enough evidence of an association between exposure to this herbicide and the following illnesses: chronic lymphocytic leukemia, soft-tissue sarcoma, non-Hodgkin’s lymphoma, Hodgkin’s disease, and chloracne.

While the acute toxicity of pesticides has been well documented, there is still little known about the adverse effects of long-term chronic exposure on human health. According to the World Health Organization (WHO), long-term exposure to pesticides could increase the risk of developmental and reproductive disorders, immune-system disruption, endocrine disruption, impaired nervous-system function, and development of certain types of cancers, such as breast cancer [52]. Attempts to establish correlations between the effects of pesticides on human health are particularly difficult because there are known sex, genetic, epigenetic, and environmental differences in the capacity to metabolize xenobiotics. Differences in susceptibility may also be affected by variations in the rate at which the xenobiotics are eliminated from the body. The adverse effects of pesticides may be related to their interactions with AhR, but they may also be partially or totally mediated by an AhR-independent signaling pathway. In both cases, specific windows of exposure to pesticides during a lifetime can have different outcomes thus complicating the interpretation of epidemiological studies. In addition, genetic differences in the properties of AhR are known to exist in human populations, and polymorphisms in cytochrome P450 enzymes have been associated with increased susceptibility to different cancers [53].

Consequently, this demonstrates the challenges as well as the importance of better understanding the mechanisms that underline the crosstalk that exist between dioxin receptor and estrogen receptor signalling, and also rigorously test how different pollutants and pesticides affect this crosstalk.

## 5. Molecular Mechanisms of AhR and ERα Crosstalk

Interaction between the AhR and the ERα signaling pathways has been observed for several years. ERα belongs to the nuclear receptor family of transcription factors and is involved in the regulation of cellular proliferation in response to E2, for example during mammary gland development [54]. Numerous laboratories have focused their interests in studying the inhibition of the ERα signaling pathway by AhR. However, the role of ERα in the regulation of the expression of AhR target genes is less documented. Firstly, we will review the main conclusions and mechanisms proposed for the regulation of ERα by AhR. Secondly, we will discuss why the study of the differential regulation of AhR target genes (*CYP1A1* and *CYP1B1*) by ERα is important, and why this can influence mammary carcinogenesis.

## 6. AhR-Mediated Repression of the ERα Signaling Pathway

The earliest study to establish a link between pollutants and estrogen-induced cancer was carried out by Kociba and collaborators in 1978 [55]. They made the striking observation that female Sprague-Dawley rats treated with TCDD for two years developed less mammary and uterine tumors than non treated rats [55]. Ten years later, other studies showed that TCDD treatment also inhibits proliferation only in ERα positive breast cancer cell lines, and not in ERα negative cell lines [56,57]. The chemopreventive and chemotherapeutic activities of TCDD in breast carcinogenesis triggered the interest of numerous laboratories to find or to develop AhR agonists that possess the antiestrogenic activity of TCDD, but without its acute toxicity. These AhR agonists are called Selective AhR Modulator (SAhRM). One example of such a SAhRM is the DIM, an acid-catalyzed metabolite of I3C, which is a compound found in cruciferous vegetables. Chen and colleagues showed that female rats treated with DIM had a decrease in E2-dependant 7,12-dimethylbenzanthracene (DMBA)-induced mammary tumors [58]. However, DIM, additionally of AhR activation, also activates ERα by ligand-independent pathways mediated by the PKA and MAPK signaling [59], which could have deleterious effects [60].

The regulation of ERα by AhR acts at many levels, for which four different mechanisms have been proposed: firstly, studies showed that AhR and ERα interact with common transcription factors and coactivators in order to modulate transcription [61,62,63,64]. Consequently, when the two pathways are activated simultaneously, AhR and ERα compete for the binding of these factors. Secondly, AhR represses some ER target genes by binding directly their promoter. The first example to document this mechanism was discovered at the *cathepsin D* promoter where the sequence of the pentanucleotide core of the XRE is found [65,66]. The presence of the inhibitory XRE (iXRE) is necessary for the repression of *cathepsin D* by AhR. Functional iXREs were later identified in the promoter of *c-fos*, *hsp27* and *TFF1* genes [67,68,69]. Thirdly, the activation of AhR increases the degradation of ERα by the ubiquitin-proteasome pathway [19,70]. Ohtake and co-workers showed that AhR is associated with an E3-ubiquitine ligase complex, which is proposed to be involved in ERα degradation [15]. Additionally, using an Estrogen response element (ERE) placed upstream of a luciferase reporter, they showed that the presence of MG132 (a proteasome inhibitor) abrogates the repression elicited by AhR in a ligand-specific manner. However, this result is challenged by other studies showing that MG132 affects the mRNA levels of the ERα target genes, even in the absence of AhR ligands [71,72]. The discrepancies between these results raise a major concern regarding data obtained with reporter constructs. Nevertheless, activated-AhR and E2-mediated degradation of ERα borrows two different pathways, which could explain why one is necessary for ERα target genes expression and the other one inhibits it [15]. Fourthly, in breast cancer cells, TCDD induces the expression of *CYP1A1* and *CYP1B1*, which encode enzymes that convert E2 into catecholestrogens [41,42]. The expression of these enzymes leads to an increase in E2 metabolism and a reduction of intracellular E2 concentration. Although, this mechanism may contribute in the repression of the ERα signaling pathway by AhR, it is not sufficient to explain all the cellular effects that are observed. Indeed, the repression of *cathepsin D* expression mediated by TCDD occurs very quickly after E2 treatment, and at this time point, *CYP1A1* is not yet induced [65]. Moreover, in rats treated with TCDD, the level of E2 circulating in the blood is not affected [73]. In conclusion, the pathway involving E2 metabolizing enzymes is not necessary for the inhibitory crosstalk observed between AhR and ERα.

## 7. ERα-Mediated Repression of AhR Target Genes

In mammary tissues, *CYP1A1* and *CYP1B1* are the two most induced AhR target genes after TCDD treatment. As previously mentioned, the enzymes encoded by these two genes are involved in E2 metabolism: CYP1A1 converts E2 in 2-OHE_2_, while CYP1B1 converts E2 in 4-OHE_2_, one of the most genotoxic estrogen metabolites. Interestingly, ERα specifically represses *CYP1A1* gene expression, but not *CYP1B1*. We propose that action of ERα may constitute another mechanism by which it promotes carcinogenesis.

**Figure 2:**
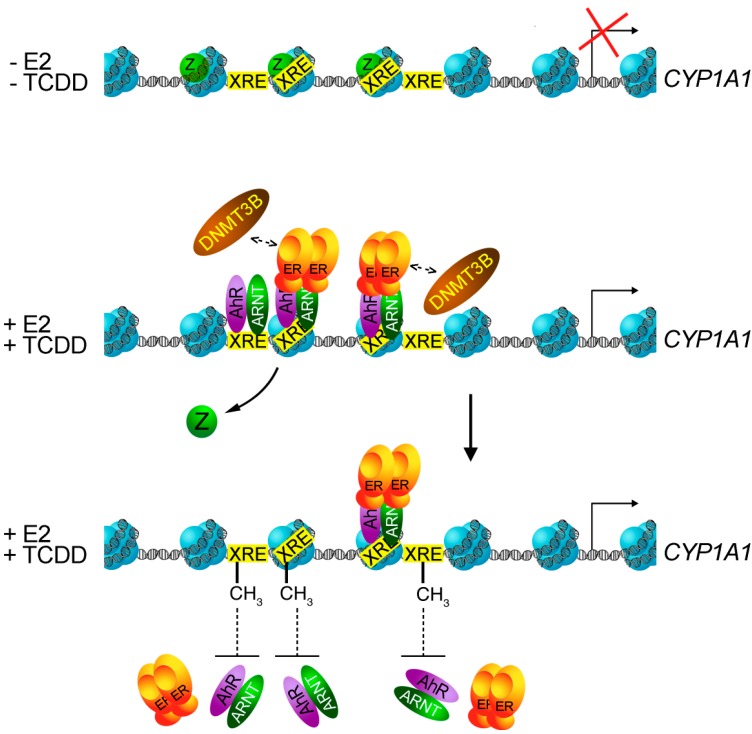
Proposed model for the regulation of the *CYP1A1* gene by ERα. In the absence of estradiol and 2,3,7,8-tetrachlorodibenzo-*p*-dioxin (TCDD), *CYP1A1* is not expressed and H2A.Z is present in its promoter. In the presence of both ligands, H2A.Z is removed and AhR/Arnt/ERα is recruited to the *CYP1A1* promoter. ERα displaces AhR/Arnt by promoting DNA methylation on the XREs in the *CYP1A1* promoter, thus resulting in less AhR activating surfaces available to stimulate *CYP1A1* expression than in presence of TCDD alone.

Our laboratory has focused efforts to unravel the mechanism(s) involved in the *CYP1A1* gene repression by ERα in breast cancer cell lines. We discovered two new key players in the regulation of *CYP1A1* expression in ERα positive cell lines: the histone variant H2A.Z and the DNA methyltransferase Dnmt3B. Depletion of H2A.Z in MCF7 cells triggers DNA methylation of AhR binding sites in the *CYP1A1* promoter, and thus affects *CYP1A1* induction in the presence of AhR agonists. In cell lines which do not express ERα, the absence of H2A.Z has no effect on the induction of *CYP1A1* [60]. Previous work from the Henikoff laboratory showed that H2A.Z antagonizes DNA methylation in *Arabidopsis thaliana* [74] and a similar conclusion has been drawn in a murine model [75]. The same mechanism appears to be true at the *CYP1A1* promoter in the presence of ERα. Moreover, we showed that ERα recruitment to the *CYP1A1* promoter in presence of TCDD and E2 decreases AhR binding at its XREs, which leads to two times less induction of *CYP1A1* mRNA level than in the presence of TCDD alone. We also discovered that the inhibition of DNA methylation, by either 5-azacytidine treatment or by depleting Dnmt3B, impairs specific inhibition of *CYP1A1* by ERα [60]. Taken together, these new findings suggest that DNA methylation plays a central function in the regulation of the *CYP1A1* expression in the presence of ERα. A model is proposed for this mechanism in Figure 2.

## 8. Conclusions

Industrialized countries generate more and more pesticides and pollutants. However, there is a general concern in fabricating useful chemicals that are not “as toxic” as older generation pesticides. Nonetheless, there is no way to tell how the pesticides used today in our agricultural practices will influence our health on a long-term basis. It is clear that even though a particular pollutant may not present a high health risk—probably based on toxicological tests performed with laboratory animals—it may still significantly perturb endocrine systems, and lead to the generation of genotoxic metabolites. We believe that genes involved in the regulation of the crosstalk that exists between the dioxin receptor and estrogen receptor signaling could become important molecular sensors, or biomarkers, to assess potential long-term effects of pesticides on certain forms of cancer.
